# The use of machine learning in transarterial chemoembolisation/transarterial embolisation for patients with intermediate-stage hepatocellular carcinoma: a systematic review

**DOI:** 10.1007/s11547-025-02013-y

**Published:** 2025-05-03

**Authors:** Lakshya Soni, Jasen Soopramanien, Amish Acharya, Hutan Ashrafian, Stamatia Giannarou, Nicos Fotiadis, Ara Darzi

**Affiliations:** 1 Institute of Global Health Innovation, Imperial College London, London, UK; 2 Royal Marsden Hospital, London, UK; 3 Queen’s Medical Centre, Nottingham, UK; 4 Hamlyn Centre for Robotic Surgery, Imperial College London, London, UK; 5 Institute of Cancer Research, London, UK

**Keywords:** Carcinoma, Hepatocellular, Chemoembolization, Therapeutic, Machine Learning, Image Interpretation, Computer-Assisted, Interventional Radiology

## Abstract

Hepatocellular carcinoma (HCC) is one of the leading causes of cancer-related deaths worldwide. Intermediate-stage HCC is often treated with either transcatheter arterial chemoembolisation (TACE) or transcatheter arterial embolisation (TAE). Integrating machine learning (ML) offers the possibility of improving treatment outcomes through enhanced patient selection. This systematic review evaluates the effectiveness of ML models in improving the precision and efficacy of both TACE and TAE for intermediate-stage HCC. A comprehensive search of PubMed, EMBASE, Web of Science, and Cochrane Library databases was conducted for studies applying ML models to TACE and TAE in patients with intermediate-stage HCC. Seven studies involving 4,017 patients were included. All included studies were from China. Various ML models, including deep learning and radiomics, were used to predict treatment response, yielding a high predictive accuracy (AUC 0.90). However, study heterogeneity limited comparisons. While ML shows potential in predicting treatment outcomes, further research with standardised protocols and larger, multi-centre trials is needed for clinical integration.

**Supplementary Information:**

The online version contains supplementary material available at 10.1007/s11547-025-02013-y.

## Introduction

In 2020, there were an estimated 906,000 new cases of primary liver cancer and 830,000 associated deaths. 80% of these cases were due to hepatocellular carcinoma (HCC), making it the most common primary liver cancer [1]. Unfortunately, its incidence continues to rise with the increasing worldwide prominence of its main risk factors, including alcohol-related liver disease, metabolic-associated liver disease (MASLD—formerly known as non-alcoholic fatty liver disease), hepatitis B and C virus infections (HBV & HCV) and aflatoxin exposure [2–14]. After initiation of the pathophysiological process from one of these triggers, a cascade of hepatic inflammation, fibrosis and aberrant hepatocyte regeneration ensues that eventually results in liver cirrhosis. One-third of these patients with liver cirrhosis will go on to develop HCC in their lifetime [15–17].

HCC treatment is guided by the Barcelona Clinic Liver Cancer (BCLC) staging system.

The BCLC staging system has four main classifications:

0—Very early stage. Single lesion less than 2 cm in size with preserved liver function.

A—Early stage. Single or fewer than 3 nodules, each less than 3 cm in size, with preserved liver function.

B—Intermediate stage. Multinodular with preserved liver function.

C—Advanced stage. Portal invasion + /− extra-hepatic spread with preserved liver function.

D – Terminal stage. Any tumour burden with end-stage liver function.

Treatment options include ablation, resection, transplantation, and systemic therapies such as atezolizumab/bevacizumab and tremelimumab/durvalumab for advanced stages [18–32]. Transcatheter arterial chemoembolisation (TACE) remains the standard for intermediate-stage HCC and is the primary treatment recommendation for patients with BCLC Stage B with preserved liver function.

TACE is the gold standard for patients with intermediate-stage HCC with relatively preserved liver function. It has been shown to improve median survival from 16 to 20 months compared to best supportive care [30].

TACE refers to the administration of an emulsion made with chemotherapeutic drugs mixed with iodised oil, followed by embolisation of the blood vessel. It is administered via an intra-arterial catheter directly to the treatment site, near the vessels that supply the HCC [33].

Despite its benefits, several limitations of the TACE procedure have been highlighted. Inconsistency in the technique, treatment schedules and response to treatment are all impacted by the variation in cellular composition, metabolism and anatomy, which varies naturally between different patients, making it hard to identify which patients would benefit from TACE. Some studies have even shown that a proportion of patients may need up to 3 repeated TACE procedures before achieving a clinically relevant response, inevitably considerably increasing the associated risk of liver injury, iatrogenic complications and chemotherapy-associated complications with every procedure [34]. Another limitation is seen with the variable dosing of the chemotherapy agents used. The specific dose is often calculated based on the location and uptake of the agent but is often not very accurate. To accommodate this, drug-eluting beads (DEB-TACE) have recently gained favour, as they increase the pharmacokinetic profile of TACE and provide consistency that is not seen with TACE alone [30].

Artificial Intelligence (AI), particularly convolutional neural networks (CNNs), is increasingly applied in hepatology for diagnostic andprognostic purposes [35–37]. Machine learning (ML), a subset of AI, enables computers to learn from data and improve task performance without explicit programming. Supervised, unsupervised and reinforcement learning allow systems to analyse labelled or unlabelled data and optimise decision-making processes. In radiology, ML has enhanced diagnostic accuracy, treatment planning and patient outcomes. For instance, ML algorithms improve the detection and characterisation of liver lesions by analysing imaging data [36], assisting in the planning of complex interventions [38] and predicting patient outcomes to personalise treatments [35–37]. ML algorithms have been shown to improve the detection and characterisation of liver lesions in ultrasound imaging with an accuracy of up to 96.6% [39]. However, AI's role in optimising TACE procedures is not well-documented, but the initial signs are promising.

Deep learning approaches in interventional radiology have identified catheters and guidewires during angiography [40], which may lead to real-time road-mapping of vasculature without the need for contrast [41]. A similar real-life use case currently exists for planning and guiding prostate artery embolisation using virtual injection software to map vasculature and plan the embolisation route before overlaying it as a 3D roadmap, augmenting live fluoroscopy images to guide microcatheter navigation [42]. Some new reviews demonstrate its use in the treatment of HCC with transcatheter arterial radioembolisation [43]; however, there is a paucity of reviews examining its application more broadly in the context of TACE.

Radiomics, a quantitative approach to extracting features from medical imaging, offers significant promise in analysing tumour heterogeneity and predicting treatment outcomes. In the context of TACE for intermediate-stage HCC, the integration of radiomic features—such as the grey-level co-occurrence matrix (GLCM) and wavelet-transformed metrics—has demonstrated utility in identifying tumour morphology and pathology [44]. However, a critical limitation in the current literature is the lack of uniformity in radiomic feature extraction protocols. Standardising these methods under the Image Biomarker Standardisation Initiative (IBSI) is essential to ensure reproducibility and comparability across studies [45].

Despite instances of AI being used in the diagnosis and prognostication of HCC, no literature currently available summarises its use during the TACE procedure as a treatment modality and analyses its effectiveness. This review looks to outline the current uses of AI during TACE procedures, critically evaluate its efficacy through these studies and examine important gaps within the existing literature.

## Methods

This study was prospectively registered on PROSPERO [46], the international prospective register of systematic reviews (registration number CRD42023478356). It was carried out and reported per the 2019 PRISMA (Preferred Reporting Items for Systematic Reviews and Meta-Analyses) statement [47].

### Search strategy

A comprehensive literature search was performed on Medline, Embase, Scopus and the CENTRAL databases. This included all articles published up to and including June 1, 2024. The full search terms are in the supplementary material (S1).

### Study selection

The retrieved studies were uploaded onto the Covidence platform (Covidence, Melbourne, Australia), where screening was performed by two independent authors (LS and JS). Disagreements were discussed with an independent third author (AA) until a consensus was reached. Duplicates were screened and removed accordingly. Titles and abstracts were subsequently screened for relevance, with irrelevant studies excluded at this stage. Full-text reviews were then performed against the inclusion and exclusion criteria – disagreements relating to eligibility were discussed with another independent author (AA), who made the final decision. All eligible studies met the inclusion and exclusion criteria.

### Inclusion and exclusion criteria

All studies which investigated patients with Barcelona Clinic Live Cancer (BCLC) Stage B or equivalent and used machine learning for image guidance during TACE/TAE procedures were included. Given that the European Association for the Study of the Liver (EASL) guidelines recommended the use of TACE in patients who had BCLC Stage B, this was chosen as part of the inclusion criteria as it followed widely accepted international guidelines [5]. Given the experimental nature of ML-informed image guidance in TACE, randomised controlled trials, retrospective studies and case series were included. However, review articles or those in which only the abstract was available were excluded. Reported outcomes of included studies related to change in procedural time, change in radiation/contrast dose, progression-free survival, overall survival, quantitative EASL (qEASL) criteria, tumour size or the mRECIST criteria. Studies which involved patients with portal invasion/extra-hepatic spread, liver metastases or other types of liver cancer, or the use of other treatment modalities before the incident TACE procedure (e.g. surgical resection or systemic therapy) were excluded. No language restriction or temporal restriction was placed upon the search.

### Data extraction

Two authors (LS and JS) independently extracted and uploaded data onto a standard spreadsheet. For each study, the following data were extracted: study details (institution, corresponding author details, year of publication, country of the study), method design (design, grouping, algorithm type), population characteristics (age, ethnicity, gender, BCLC stage, Child–Pugh Stage, ALP, ALT, AST, GGT, AFP levels, number of intrahepatic tumours, diameter and volume of tumours and number of participants) and intervention details (imaging modality, TACE/TAE procedure used and volume of chemoembolisation used if described). The search included studies of all languages, with contact made to authors for further information on publication and data as required.

### Study quality assessment

The overall risk of bias and applicability was independently assessed using the Quality Assessment of Diagnostic Accuracy Studies 2 score (QUADAS-2) [48]. According to the scoring system, each study was scored as low, low-moderate, moderate, moderate-high or high risk of bias. Although QUADAS-2 was not specifically designed to analyse ML diagnostic accuracy studies, it has been used in similar reviews [49].

### Statistical analysis

A bivariate model for diagnostic meta-analysis was used to calculate the estimate for sensitivity, specificity and area under the curve (AUC) data. The DerSimonian and Laird random-effects modelling was used to calculate variability within and between studies [50]. The study averages and 95% confidence intervals were calculated for each study and presented in a forest plot. The heterogeneity of the studies was calculated using *I*^*2*^. Publication bias was assessed using a funnel plot. Standard errors and effect size were calculated using standard formulae. SPSS version 29.0.2.0 was used for all statistical analysis.

The pooled aggregation of the AUC values was calculated as the weighted average relative to the total number of participants. Only the implied best test AUC value was used in each study unless multiple machine learning techniques were used: in this case, multiple AUC values were included and specified in the forest plot. Two independent reviewers decided the best AUC value, with a third senior reviewer presiding over any areas of contention.

## Results

**Fig. 1:**
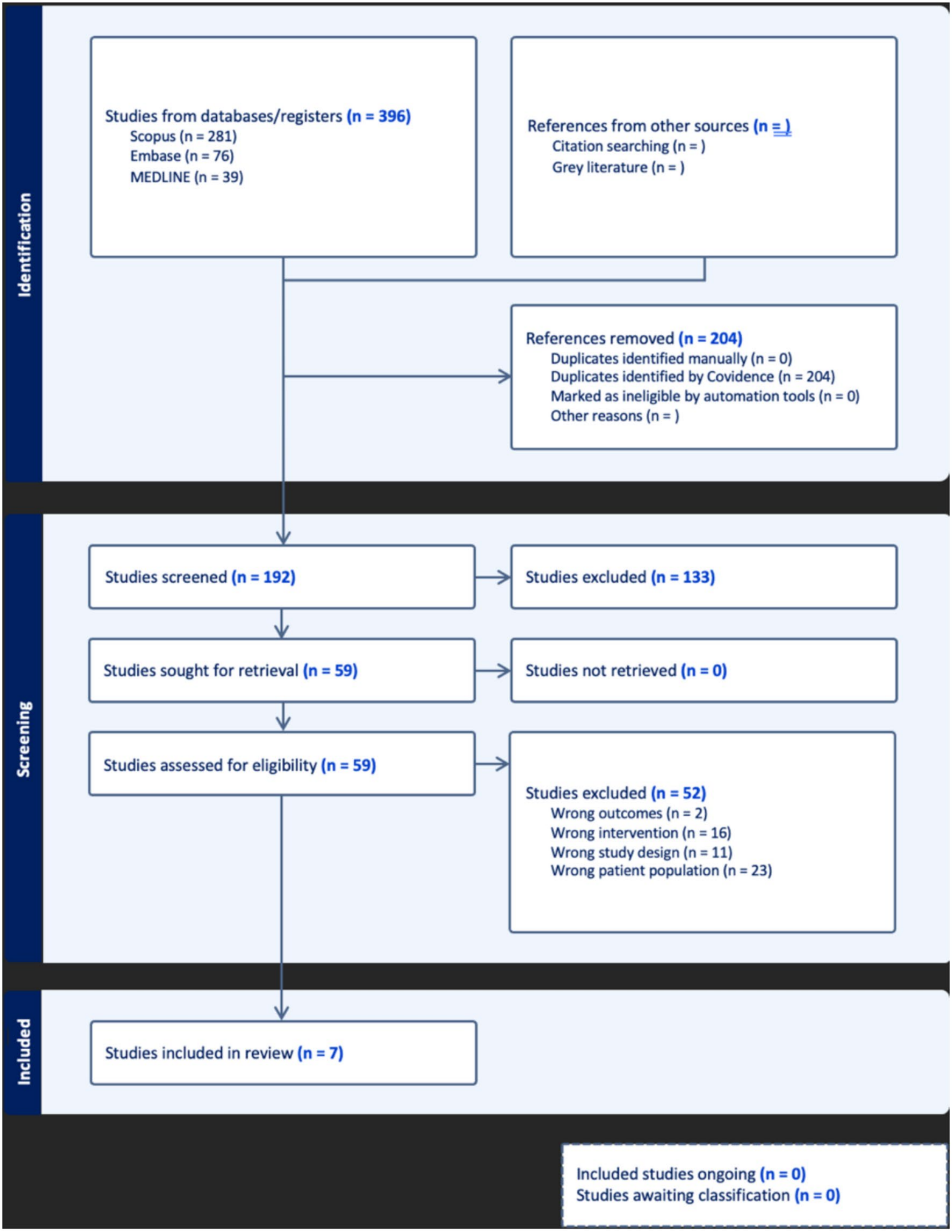
PRISMA diagram demonstrating literature search and screening

The initial screening process yielded 396 studies. A PRISMA diagram in Fig. 1 outlines the number of studies excluded at each stage. After 204 duplicates were removed, 192 studies were retained, investigating the role of machine learning in TACE. During the abstract screening stage, 133 studies were excluded as they did not meet the inclusion criteria for the review. 59 studies underwent full-text review and 37 were excluded as they were prospective studies, including drug-eluting beads or included patients with metastatic disease. Therefore, the systematic review and meta-analysis included 7 studies that met the criteria, as shown in Tables 1 and 2. Detailed radiomic feature extraction, where applicable, can be found in the supplementary materials (S2).

**Table 1:** Table outlining demographics of included papers, patient demographics, qualitative analysis and outcome measures

Paper Label	Authors	Journal	Language	Country	Date of Publication	Number of Participants	Gender	Age	Risk of Bias	Outcome Measure
1	Jie Peng, Shuai Kang, Zhengyuan Ning, Hangxia Deng, Jingxian Shen, Yikai Xu, Jing Zhang, Wei Zhao, Xinling Li, Wuxing Gong, Jinhua Huang and Li Liu	European Radiology	English	China	Jul-19	789	Males: 89 (11%) Females: 700 (89%)	≤ 60 = 555 (70%) > 60 = 234 (30%)	Low	Objective response to TACE
2	Jie Peng, Jinhua Huang, Guijia Huang and Jing Zhang	Frontiers in Oncology	English	China	Oct-21	310	Males: 273 (88%) Females: 37 (12%)	≤ 60: 214 (69%) > 60: 96 (31%)	Low-Moderate	Objective response to TACE
3	Zhongqi Sun, Zhongxing Shi, Yanjie Xin, Sheng Zhao, Hao Jiang, Dandan Wang, Linhan Zhang, Ziao Wang, Yanmei Dai and Huijie Jiang	Frontiers in Bioengineering and Biotechnology	English	China	Nov-21	399	Males: 287 (72%) Females: 112 (28%)	≤ 60 = 256 (64%) > 60 = 143 (36%)	Low	Objective response to TACE
4	Jie Peng, Fangyang Lu, Jinhua Huang, Jing Zhang, Wuxing Gong, Yong Hu and Jun Wang	Frontiers in Oncology	English	China	Oct-22	313	Males: 272 (66%) Females: 41 (34%)	≤ 60 = 211 (67%) > 60 = 102 (33%)	Low	Objective response to TACE Overall survival at 3 years post-TACE where n = 46
5	Lu Zhang, Zhe Jin, Chen Li, Zicong He, Bin Zhang, Qiuying Chen, Jingjing You, Xiao Ma, Hui Shen, Fei Wang, Lingeng Wu, Cunwen Ma & Shuixing Zhang	La radiologia medica	English	China	Feb-24	367	Males: 143 (39%) Females: 224 (61%)	58	Low	Objective response to TACE
6	Liu Lulu, Yang Hong, Shao Guoliang, Fan Linyin, Yang Yongbo, Pang Peipei, Chen Yuanjun	Chinese Journal of Radiology	Chinese	China	Sep-18	81	Male: 66 (81%) Female: 15 (19%)	63	Low-moderate	Overall survival at 3 years post-TACE
7	Yaying Chen, Yanhong Shi, Ruiqi Wang, Xuewen Wang, Qin Lin, Yan Huang, Erqian Shao, Yan Pan, Shanshan Huang, Linbin Lu, Xiong Chen	Journal of Cancer	English	China	Feb-24	1758	Males: 1144 (65%) Females: 614 (35%)	53.2	Low	Overall survival at 3 years post-TACE

**Table 2:** Table outlining ML model, segmentation software, radiomic extraction tool, compliance with IBSI standards, CT equipment, dosage, resolution, radiomic features, other clinical inputs used in algorithm development and best test AUC value

Paper label	ML model used	Segmentation tool used	Radiomic extraction tool	IBSI compliance	CT equipment	CT dosage	CT Spatial resolution	Radiomic features	Other inputs	AUC value	
1	GhostNet	ITK-SNAP	Not Stated	Not Stated	SOMATOM (Siemens Medical Systems, Germany) Brilliance 256-iCT (Philips Healthcare, USA)	120 kVp, Automatic mAs	5 mm slice thickness—> standardised as 1 mm slices	GLCM, wavelet-transformed features, tumour size, intensity-based features	None included	0.964	
2	LASSO	ITK-SNAP	PyRadiomics	Yes	SOMATOM (Siemens Medical Systems, Germany) Brilliance 256-iCT (Philips Healthcare, USA)	120 kVp, non-specified mAs	5 mm slice thickness—> standardised as 1 mm slices	Shape descriptors, GLCM, wavelet-transformed features, intensity-based features	Tumour size, AFP, Number of tumours	Linear: 0.763 Logistic: 0.781 SVM: 0.765 GBM: 0.81 RF: 0.964 DL: 0.972 DL + Tumour size: 0.976	DL + Linear: 0.986 DL + Logistic: 0.987 DL + SVM: 0.980 DL + GBM: 0.982 DL + RF: 0.994
3	ResNet50	Dr. wise AI	Dr. Wise AI	No	64-detector row (GE Healthcare, United States)	120 kVp, 250 mAs	5 mm slice thickness	GLCM, first-order features (mean, variance), tumour shape descriptors	NLR, PLR, SII, SIRI	0.98	
4	In-house LLM (based on TensorFlow2.0)	ITK-SNAP	PyRadiomics	Yes	SOMATOM (Siemens Medical Systems, Germany) Brilliance 256-iCT (Philips Healthcare, USA)	120 kVp, non-specified mAs	5 mm slice thickness—> standardised as 1 mm slices	GLCM, GLRLM, tumour size descriptors, intensity-based features (mean, entropy), wavelet-transformed features	None included	0.911 0.64	
5	SHAP	Not Specified	Not Stated	Not Stated	GE LightSpeed Ultra 8 (GE Healthcare, Japan 64-channel (LightSpeed VCT, GE Medical Systems, USA)	120 kVp, 240 mAs	1-2 mm slice thickness	No radiomic features; focuses on AER, PER, APR	ALBI grade, AFP, C-Reactive Protein	LR Clinical: 0.567 SVM Clinical: 0.603 RF Clinical: 0.549	LR Combined: 0.611 SVM Combined: 0.714 RF Combined: 0.800
6	LASSO	ITK-SNAP	AI-Kit	No	German Siemens Definition Flash 64-slice CT scan	120 kVp, 250 mAs	5 mm slice thickness	GLCM, GLRLM, tumour volume, histogram features (mean, variance, skewness, kurtosis)	None included	0.75	
7	deepHAP IV model	Not Specified	Not Stated	Not Stated	Not specified	Not specified	Not specified	No radiomic features	Logarithmic AFP, ALB, TBL, Tumour Size	0.7	

*IBSI* Image Biomarker Standardisation Initiative, *GLCM* Grey-Level Co-occurrence Matrix, *GLRLM* Grey-Level Run Length Matrix, *NLR* Neutrophil-to-Lymphocyte Ratio, *PLR* Platelet to Lymphocyte Ratio, *SII* Systemic Immune Inflammation Index, *SIRI* Systemic Inflammation Response Index, *AFP* Alpha-fetoprotein, *ALBI* Albumin–Bilirubin Ratio, *SVM* Support Vector Machine, *GBM* Gradient Boosting Machine, *RF* Random Forest, *LR* Logistical Regression, *DL* Deep Learning, *AER* Arterial Enhancement Ratio, *PER* Portal Venous Enhancement Ratio, *APR* Arterial Portal Venous Ratio, *LLM* Large Language Model, *ALB* Albumin, *TBL* Total Bilirubin

### Overview of studies

In total, 4017 participants were included across 7 studies. The studies were split into 2 groups, according to the outcome measured in each study. Group A looks at the objective response [(OR), defined as complete response and partial response post-TACE treatment, in line with the mRECIST criteria)], and Group B looks at the 3-year overall survival (OS). The characteristics and number of participants in each study are included in Table 1. All studies implemented machine learning models to improve predictability of outcomes post-TACE procedure. 2 studies implemented Least Absolute Shrinkage and Selection Operator (LASSO), 1 study used GhostNet, 1 study used ResNet50, 1 study used deepHAP IV, 1 study used Shapley additive explanation (SHAP) and 1 study developed their own language learning model based on TensorFlow 2.0 training. All studies reported receiver operating characteristic (ROC) analysis with the area under the curve (AUC) included as a measure of the accuracy of the ML model to predict either objective response to TACE or overall survival after 3 years.

Chen et al. [51] and Peng et al. [52] were the only studies to provide data on overall survival and progression-free survival. While all studies based their AUC of ROC on true positive, true-negative, false-positive and false-negative data, only Sun et al. [53] explicitly stated these data. Finally, there was still some discrepancy in the reporting of AUC values, with one study opting to provide AUC values according to the mRECIST outcome [54], while all others reported the overall AUC value according to the cohort. For this paper, we used a pooled weighted average AUC to calculate the objective response.

All studies demonstrated strong AUC results to indicate strong predictive values of each model.

### Outcomes

#### Meta-analysis

**Fig. 2:**
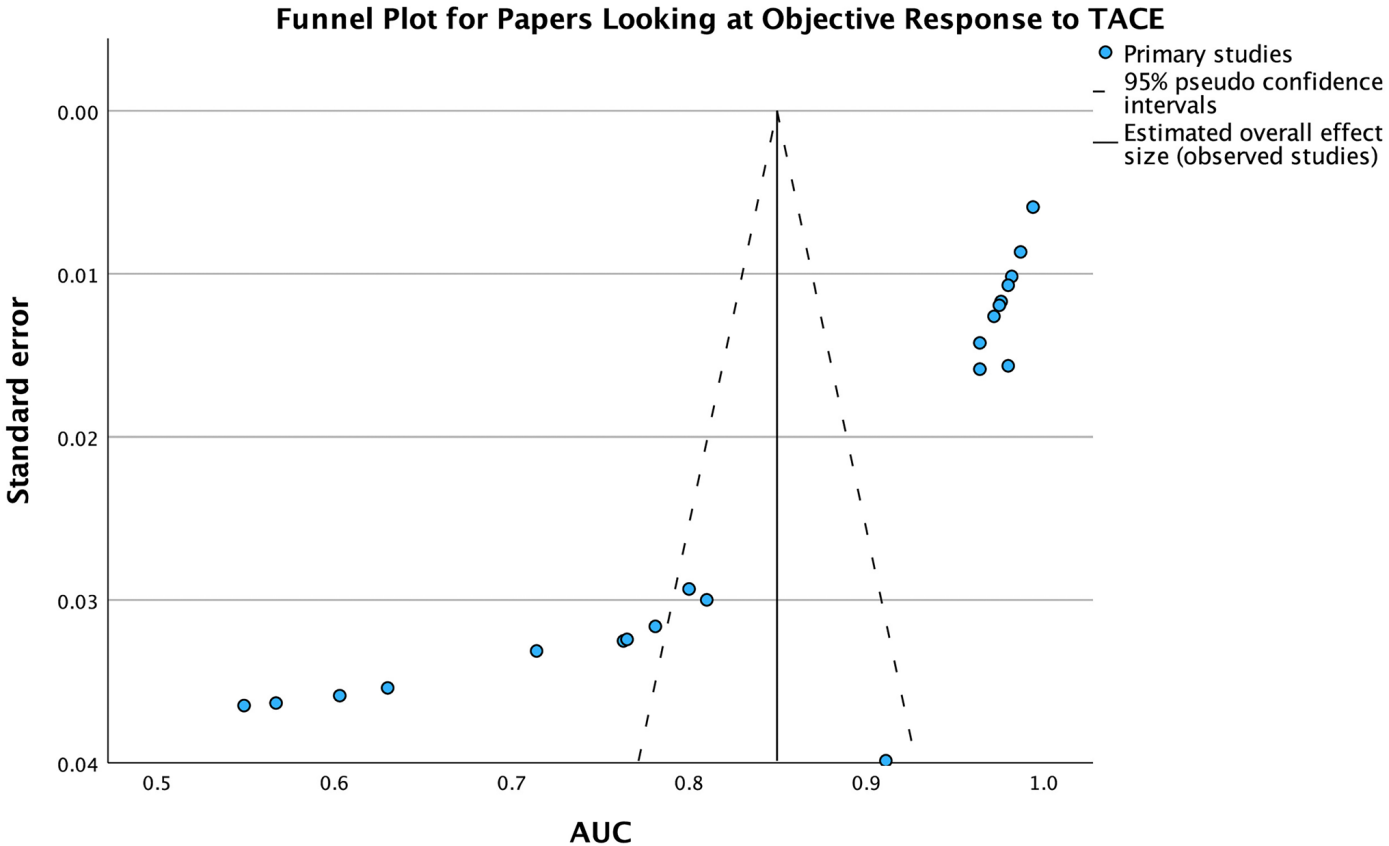
Funnel plot of papers which looked at OR to TACE

The meta-analysis included individual participant cohorts as test cohorts or validation cohorts, where the test cohort was not specified in the particular study. The papers were divided into two groups based on the outcome: OS at 3 years and OR to TACE. The number of participants in each cohort ranged from 17 to 562 (Fig. 2).

**Fig. 3:**
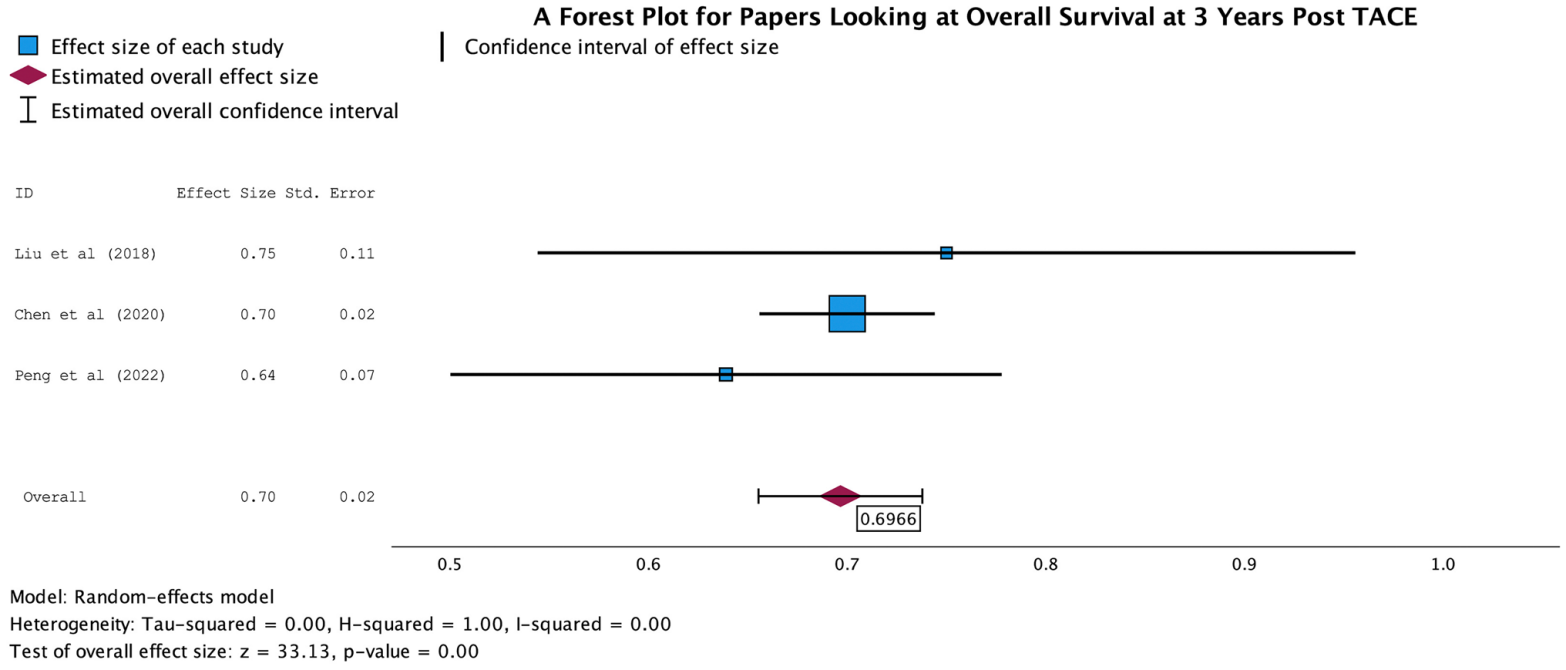
Forest plot of papers which looked at OS at 3 years post-TACE

**Fig. 4:**
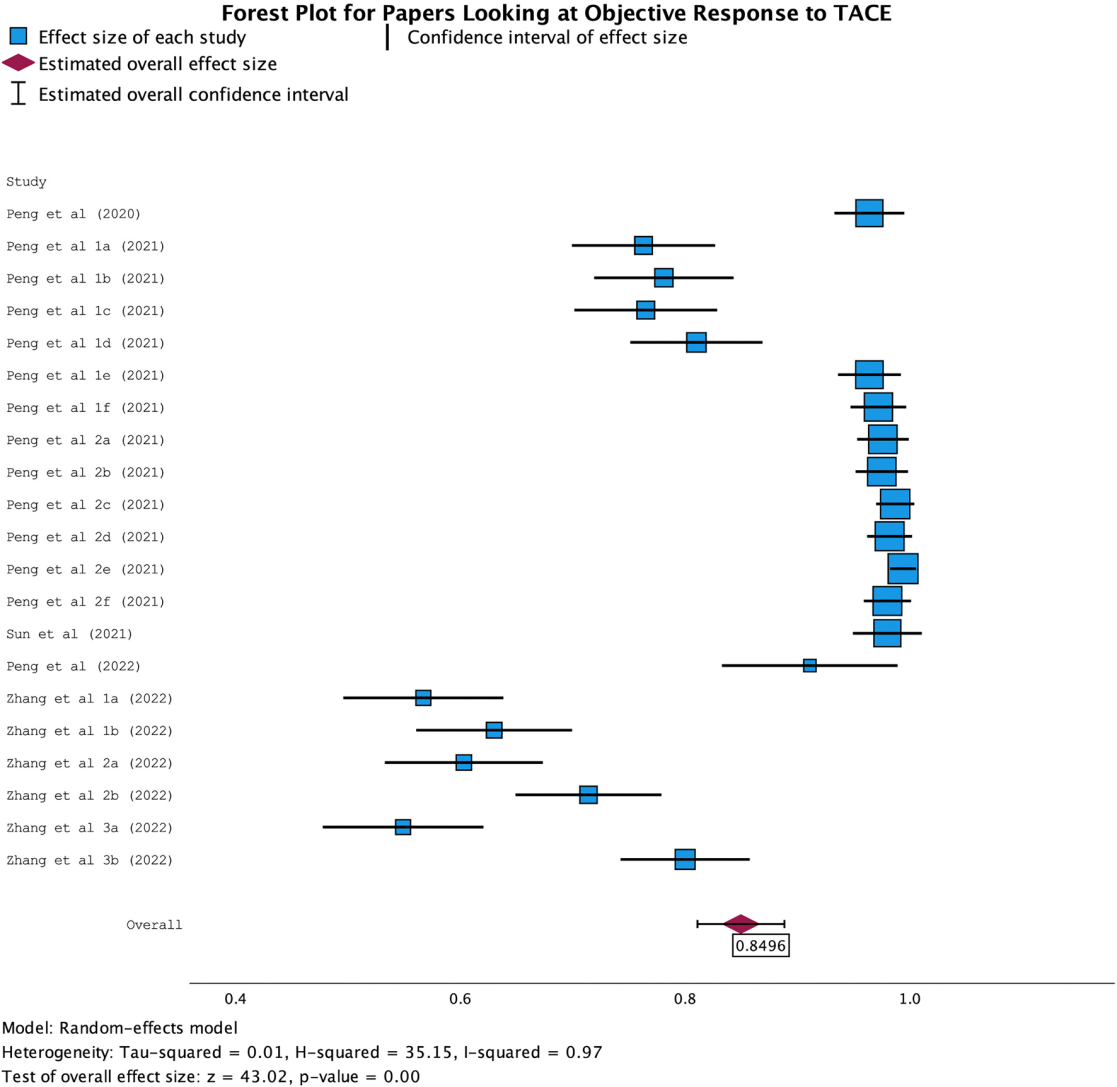
Forest plot of papers which looked at OR to TACE

The AUC was based on the ability of the ML algorithms to predict outcomes based on the pre-TACE imaging according to the mRECIST criteria. The AUC values ranged from 0.55 to 0.98 across the cohorts, demonstrating a varied but predominantly strong predictive value. Overall, in the meta-analysis, there was an overall AUC of 0.6966 for the papers predicting the overall survival at 3 years (Fig. 3) and an overall AUC of 0.8496 for papers predicting the OR to TACE (Fig. 4), with small confidence intervals reaffirming the significant findings. This demonstrates good predictive value when looking at the OR to TACE and can help categorise patients into responders and non-responders. This was lower in papers predicting OS at 3 years, suggesting less clinical utility.

Some studies found that tumour size was directly related to the prediction of post-TACE outcomes [55, 56], whilst other studies used supplemental information that augmented their results, including blood test results to form an inflammatory burden index [53], radiomics features to identify pathological components to tumours on CT imaging [52, 54], alpha-fetoprotein levels [55].

#### Quality assessment

**Fig. 5:**
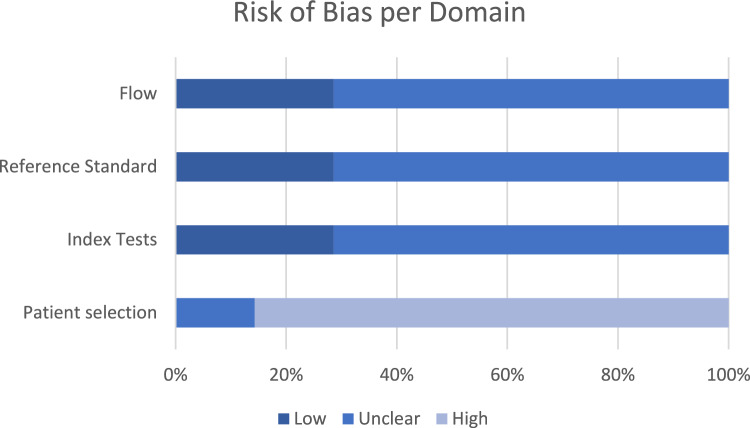
Graph displaying the percentage of studies with varying degrees of bias for each of the four QUADAS-2 domains

**Fig. 6:**
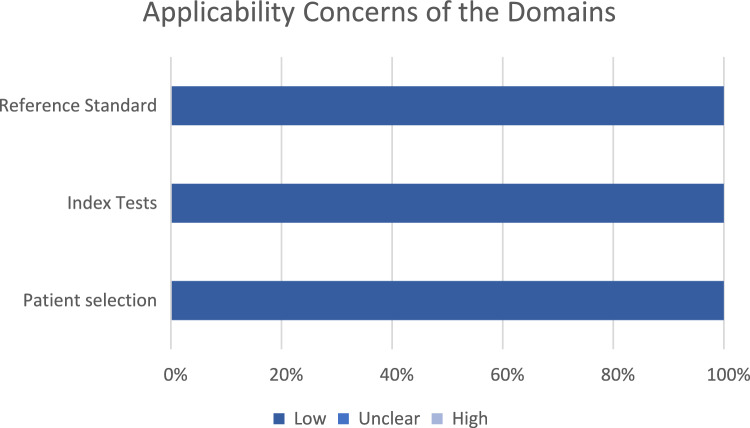
Graph displaying the percentage of studies of varying applicability for three of the four QUADAS-2 domains

The risk of bias and applicability was assessed using QUADAS-2: the results can be seen in Figs. 5 and 6 [48]. Overall, the quality of the included studies generated a low risk of bias. Most studies showed a low risk of bias in domains relating to index tests, reference standards, flow and timing. There was some lack of clarity regarding the sampling mechanisms implemented in 4 studies [52–54, 57]. One study [55] raised high concerns relating to the sampling mechanism due to questionable reasons for exclusion criteria, including missing data for appropriate candidates and loss to follow-up with no apparent explanation. One paper [56] lacked sufficient information on the blinding of the index test and reference standard interpretations.

The risk of bias was noted with the involvement of radiologists, who were invited to segment regions of interest in the CT images. Two studies did not clarify that the radiologists were blinded to the treatment outcome, raising concerns of potential bias.

A risk of publication bias must be considered in this review due to the lack of ongoing trials and grey literature included.

In the meta-analysis, the funnel plot and Egger’s regression-based test indicate significant publication bias for papers looking at objective response to TACE. The funnel plot in Fig. 2 shows asymmetry, with more studies reporting higher AUC values. Egger’s test confirms this bias with a significant intercept (coefficient = − 3.612, SE = 0.1328, t = − 27.199). Despite this, the AUC analysis demonstrates a strong overall effect size (coefficient = 6.700, SE = 0.1865, t = 35.924). These findings suggest that the meta-analysis may be skewed by the preferential publication of studies with positive outcomes.

**Fig. 7:**
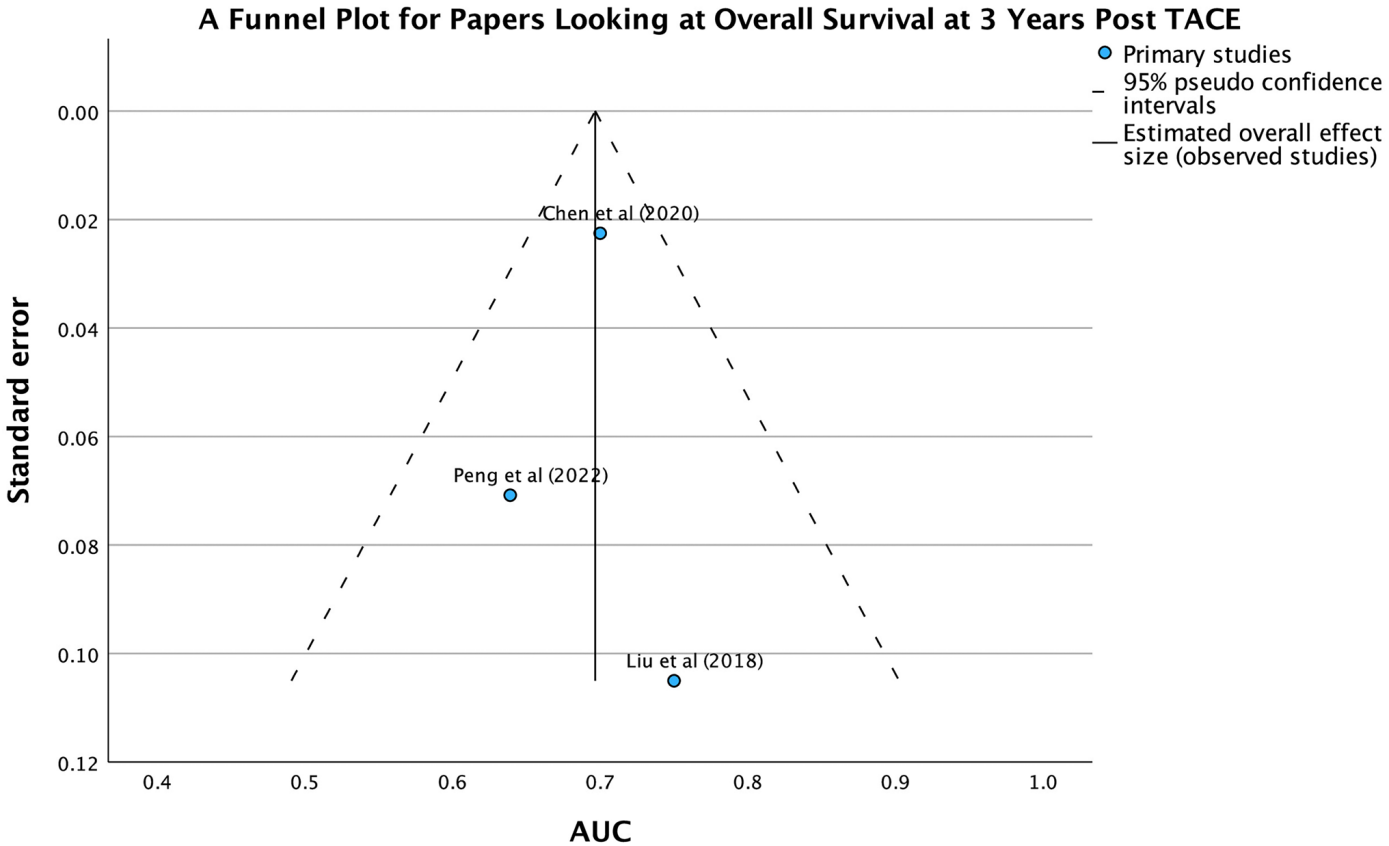
Funnel plot of papers which looked at OS at 3 years post-TACE

The funnel plot for overall survival at 3 years post-TACE, in Fig. 7, shows a roughly symmetrical distribution, suggesting minimal publication bias. However, Egger’s regression-based test reveals a significant intercept (coefficient = 0.702, SE = 0.0373, *p* = 0.034), indicating potential publication bias. Despite this, the limited number of studies may reduce the robustness of these findings.

## Discussion

This systematic review has shown that integrating machine learning into the TACE procedure has a significant impact on predicting the objective response (Group A) and overall survival (Group B) to TACE. Our meta-analysis demonstrated promising predictive outcomes, with papers in Group A having a pooled AUC of 0.8496 and Group B having a pooled AUC of 0.696. Group A demonstrated a higher strength in predicting patient outcomes, which aligns with research demonstrating that models with higher AUC values are more often clinically useful [55]. The quality assessment suggested that the findings are reliable and relevant; however, potential participant flow and selection biases should be considered when interpreting the results. These findings indicate that ML has the potential to significantly enhance the utility of TACE procedures by predicting which patients are most likely to respond well to TACE treatment.

### Overall survival

The AUC value of 0.696 suggested a weaker association between ML-associated TACE procedures and predicting overall survival at 3 years. With a value under 0.80, the clinical utility of this finding is limited [58]. However, it must be noted that only 3 of the included studies looked at this particular outcome, with 1 such study only including 81 participants. With this in mind, extrapolating conclusive lessons is challenging and further study is required to look into this association in greater detail. The inclusion of such few participants with no power calculation puts the study at risk of a type 2 error, whereby a false-negative error could arise, and a significant effect may be missed due to the underpowering of the sample.

### Objective response to TACE

Machine learning improves the pre-procedural prediction of an objective response to TACE. Based on the meta-analysis, the pooled AUC of 0.8496 suggests a strong relationship between data input to the ML programme and objective response to TACE, potentially lending itself to guiding future clinical decisions on the management of HCC. Most pertinently, it highlights patients who would fare worse with TACE and may, therefore, highlight patients who would be at greater risk of procedure failure while still being exposed to the risks of its complications. One of the most severe complications is TACE-induced liver failure, which can lead to significant morbidity and mortality. While patients are selected to minimise the risk of this, it has been reported that 60% of patients experience at least a single episode of liver decompensation during their treatment course [58].

### Integration into clinical practice

Integrating ML into the process offers key insight into the risk-to-reward balance that underpins every clinical decision. Therefore, in some cases, it may indicate switching to systemic therapies in order to preserve liver function [59]. Implementing machine learning algorithms to predict which patients will likely benefit from treatments such as TACE can help to ensure that they receive appropriate clinical management and are considered for alternative therapies at an earlier stage. This ensures that resources are appropriately directed at a time when interventional radiology is understaffed, geospatial disparities to interventional radiology care exist [60–62] and that patients are not subjected to the risks of unnecessary invasive procedures.

Integrating machine learning algorithms to predict patient responsiveness to TACE and identify those unlikely to benefit holds substantial practical implications. This approach enables the development of personalised treatment plans, enhances therapeutic outcomes and reduces unnecessary invasive interventions. The utilisation of ML for predicting treatment response signifies a significant advancement in the management of HCC, potentially transforming current clinical practices and patient pathways, as suggested by this review.

Papers in this review highlighted the positive predictive value of ML models in predicting either response to TACE or OS following TACE; however, it is important to note that these papers also highlighted the difficulties of incorporating ML and particularly DL into clinical practice. One major challenge is the requirement for large, high-quality, and diverse datasets to train robust ML models. All studies in this review were conducted in China, which limits the generalisability of findings to other populations with different demographics and clinical characteristics. This geographic limitation underscores the need for future research to include more diverse populations to ensure findings are representative and widely applicable.

Incorporating ML into clinical practice for TACE also presents significant challenges. Firstly, the development of ML algorithms requires substantial computational resources and expertise in both medical and technical domains. The “black box” nature of some ML models, particularly deep learning models, makes it difficult for clinicians to interpret the decision-making process of these algorithms, which can hinder clinical adoption.

### Standardisation of data handling

Ensuring the transparency and processes of ML models is crucial for gaining clinician trust and integrating these tools into clinical practice. Additionally, implementing ML in clinical settings demands consistent and standardised imaging protocols and data collection methods, which are often lacking, as seen by the variability amongst the included studies. This inconsistency can lead to variability in model performance and limit the reproducibility of results.

The reviewed studies varied in their use of radiomic features, ranging from texture-based measures such as GLCM to shape descriptors and wavelet-transformed features. A key observation was inconsistent compliance reporting with IBSI standards, which aim to harmonise feature extraction processes across different platforms. Adhering to these guidelines would enable greater reproducibility and facilitate multi-centre validation of ML algorithms in TACE. Future research should emphasise IBSI compliance to ensure the reliability of radiomic-based predictive models in clinical practice.

### Limitations

This review highlights several limitations, and the findings must be considered accordingly. Limitations are primarily due to the quality of the papers included. Publications are likely to favour models shown to deliver positive outcomes. While we conducted a thorough multi-database search, publication bias still exists.

This systematic review highlights several key limitations impacting the validity and generalisability of the findings. None of the studies conducted power calculations, raising concerns about sample size adequacy and the risk of type 2 errors. Additionally, a lack of standardised outcomes makes comparing results and drawing definitive conclusions about ML algorithms’ effectiveness difficult. All studies were conducted in China, limiting the geographic representation and generalizability of the findings to other populations. The demographic and clinical characteristics of Chinese patients may differ significantly from those in different regions, which could affect the applicability of the results on a global scale. This geographic limitation underscores the need for future research to include more diverse populations to ensure findings are representative and widely applicable. Lastly, this review included only seven studies, restricting the robustness of the conclusions. More diverse and numerous studies are needed to validate these findings and enhance the applicability of AI in clinical practice.

## Conclusion

The use of ML in TACE procedures for HCC shows significant promise, with high predictive accuracy reported across multiple studies. However, the current evidence is limited to the use of ML as a predictive tool to ascertain responsiveness to TACE. Currently, no literature has been shown to use ML to address the inconsistencies in the TACE technique. Future research should focus on addressing the limitations highlighted in this study and exploring the use of ML to help mitigate the inconsistencies reported as a limitation of TACE to unlock the full potential of ML in enhancing HCC treatment and improving patient outcomes. The promising results suggest a transformative potential for ML in the field, pending further validation.

## Supplementary Information

Below is the link to the electronic supplementary material.

Supplementary file1 (DOCX 16 KB)

## Declarations

### Approval committee

Not Applicable as this is a systematic review.
