# Supplementary material for: The use of machine learning in transarterial chemoembolisation/transarterial embolisation for patients with intermediate-stage hepatocellular carcinoma: a systematic review
Source: Radiol Med. 2025 May 3;130(7):1124–38. doi: 10.1007/s11547-025-02013-y (PMC12263724; doi:10.1007/s11547-025-02013-y)
Supplement: Supplementary file 1 — Supplementary file1 (DOCX 16 KB) [file 11547_2025_2013_MOESM1_ESM.docx]

# Supplementary Materials

Table 3. S1 - Search terms, databases and search date

| Database | Search Terms | Studies | Search Date |
| --- | --- | --- | --- |
| Medline | ((chemoemboli*ation or emboli*ation) and (TACE or TAE or "transcatheter arterial chemoemboli*ation" or "transcatheter arterial emboli*ation") and (machine learning or artificial intelligence or neural networks) and ("hepatocellular carcinoma" or HCC) and (CT or "computed tomography" or MRI or "magnetic resonance imaging" or radiology or "fluoroscopy")) | 17 | 01/06/2024 |
| EMBASE | ((chemoemboli*ation or emboli*ation) and (TACE or TAE or "transcatheter arterial chemoemboli*ation" or "transcatheter arterial emboli*ation") and (machine learning or artificial intelligence or neural networks) and ("hepatocellular carcinoma" or HCC) and (CT or "computed tomography" or MRI or "magnetic resonance imaging" or radiology or "fluoroscopy")) | 33 | 01/06/2024 |
| CENTRAL | (Machine Learning) AND (Hepatocellular carcinoma) AND (Transcatheter) | 0 | 01/06/2024 |
| SCOPUS | ( chemoembolization OR embolization ) AND ( tace OR tae OR "transcatheter arterial chemoembolization" OR "transcatheter arterial embolization" ) AND  ( machine AND learning OR artificial AND intelligence OR neural AND networks ) AND  ( "hepatocellular carcinoma" OR hcc ) AND ( ct OR "computed tomography" OR mri OR "magnetic resonance imaging" OR radiology OR fluoroscopy ) AND NOT genetics OR genes | 123 | 01/06/2024 |
| EThOS | (Machine Learning) AND (Transcatheter) | 0 | 01/06/2024 |

*Table 4. S2 - Table outlining papers and detailed list of radiomic features if provided*

| **Authors** | **Journal** | **Radiomics Used** |
| --- | --- | --- |
| **Jie Peng, Shuai Kang, Zhengyuan Ning, Hangxia Deng, Jingxian Shen, Yikai Xu, Jing Zhang, Wei Zhao, Xinling Li, Wuxing Gong, Jinhua Huang and Li Liu** | European Radiology | Not provided |
| **Jie Peng, Jinhua Huang, Guijia Huang and Jing Zhang** | Frontiers in Oncology | wavelet.LLL_ngtdm_Busyness wavelet.LLL_ngtdm_Complexity squareroot_ngtdm_Strength wavelet.LLL_glszm_LargeAreaLowGrayLevelEmphasis wavelet.HLL_ngtdm_Strength  wavelet.LLL_glszm_GrayLevelVariance original_ngtdm_Strength wavelet.LLL_ngtdm_Contrast wavelet.HLL_gldm_GrayLevelNonUniformity wavelet.HLL_ngtdm_Coarseness  original_ngtdm_Complexity logarithm_ngtdm_Busyness  wavelet.LLL_glrlm_GrayLevelNonUniformity wavelet.HLL_ngtdm_Complexity |
| **Zhongqi Sun, Zhongxing Shi, Yanjie Xin, Sheng Zhao, Hao Jiang, Dandan Wang, Linhan Zhang, Ziao Wang, Yanmei Dai and Huijie Jiang** | Frontiers in Bioengineering and Biotechnology | Not provided |
| **Jie Peng, Fangyang Lu, Jinhua Huang, Jing Zhang, Wuxing Gong, Yong Hu and Jun Wang** | Frontiers in Oncology | Intercept exponential_glrlm_Long Run Emphasis "exponential_glrim_Long Run Low Gray Level Emphasis exponential_glszm_Small Area High Gray Level Emphasis logarithm_first order_Skewness logarithm_glcm_Idmn" original _gldm_Dependence Variance "original_gldm_Small Dependence High Gray Level Emphasis original_glszm_Gray Level Non Uniformity original_shape_Maximum 2D Diameter Slice" original_shape_Maximum 3D Diameter original_shape_Sphericity "square_glszm_Small Area Emphasis wavelet.HHL_firstorder_Skewness" "wavelet.HHL_glcm_Cluster Prominence wavelet.HHL_glszm_Gray Level Non Uniformity wavelet.HHL_glszm_Large Area High Gray Level Emphasis wavelet. HHL_glszm_Low Gray Level Zone Emphasis wavelet.HLH_gldm_Dependence Non Uniformity Normalized wavelet.LHH_first order_Skewness" "wavelet.LHL_glszm_Large Area Low Gray Level Emphasis wavelet.LLH_first order_Median wavelet.LLH_glcm_Cluster Shade" wavelet.LLL_first order_90 Percentile wavelet.LLL_glcm_Idmn |
| **Lu Zhang, Zhe Jin, Chen Li, Zicong He, Bin Zhang, Qiuying Chen, Jingjing You, Xiao Ma, Hui Shen, Fei Wang, Lingeng Wu, Cunwen Ma & Shuixing Zhang** | La radiologia medica | Not provided |
| **Liu Lulu, Yang Hong, Shao Guoliang, Fan Linyin, Yang Yongbo, Pang Peipei, Chen Yuanjun** | Chinese Journal of Radiology | Not provided |
| **Yaying Chen, Yanhong Shi, Ruiqi Wang, Xuewen Wang, Qin Lin, Yan Huang, Erqian Shao, Yan Pan, Shanshan Huang, Linbin Lu, Xiong Chen** | Journal of Cancer | Not provided |
